# Timing of tracheostomy and patient outcomes in critically ill patients requiring extracorporeal membrane oxygenation: a single-center retrospective observational study

**DOI:** 10.1186/s40560-022-00649-w

**Published:** 2022-12-30

**Authors:** Ryota Nukiwa, Akinori Uchiyama, Aiko Tanaka, Tetsuhisa Kitamura, Ryota Sakaguchi, Yoshimitsu Shimomura, Suguru Ishigaki, Yusuke Enokidani, Tomonori Yamashita, Yukiko Koyama, Takeshi Yoshida, Natsuko Tokuhira, Naoya Iguchi, Yasushi Shintani, Shigeru Miyagawa, Yuji Fujino

**Affiliations:** 1 Department of Anesthesiology and Intensive Care Medicine, Osaka University Graduate School of Medicine, 2-15 Yamadaoka, Suita, Osaka 565-0871 Japan; 2 Department of Intensive Care, University of Fukui Hospital, Yoshida, Fukui, Japan; 3 Division of Environmental Medicine and Population Sciences, Department of Social and Environmental Medicine, Osaka University Graduate School of Medicine, Suita, Osaka Japan; 4 Department of Hematology, Kobe City Hospital Organization, Kobe City Medical Center General Hospital, Kobe, Japan; 5 Department of Pediatrics, Osaka University Graduate School of Medicine, Suita, Osaka Japan; 6 Department of General Thoracic Surgery, Osaka University Graduate School of Medicine, Suita, Osaka Japan; 7 Department of Cardiovascular Surgery, Osaka University Graduate School of Medicine, Suita, Osaka Japan

**Keywords:** Tracheostomy, Extracorporeal membrane oxygenation, Complication, Prognosis, Intensive care

## Abstract

**Background:**

Extracorporeal membrane oxygenation (ECMO) is an integral method of life support in critically ill patients with severe cardiopulmonary failure; however, such patients generally require prolonged mechanical ventilation and exhibit high mortality rates. Tracheostomy is commonly performed in patients on mechanical ventilation, and its early implementation has potential advantages for favorable patient outcomes. This study aimed to investigate the association between tracheostomy timing and patient outcomes, including mortality, in patients requiring ECMO.

**Methods:**

We conducted a single-center retrospective observational study of consecutively admitted patients who were supported by ECMO and underwent tracheostomy during intensive care unit (ICU) admission at a tertiary care center from April 2014 until December 2021. The primary outcome was hospital mortality. Using the quartiles of tracheostomy timing, the patients were classified into four groups for comparison. The association between the quartiles of tracheostomy timing and mortality was explored using multivariable logistic regression models.

**Results:**

Of the 293 patients treated with ECMO, 98 eligible patients were divided into quartiles 1 (≤ 15 days), quartile 2:16–19 days, quartile 3:20–26 days, and 4 (> 26 days). All patients underwent surgical tracheostomy and 35 patients underwent tracheostomy during ECMO. The complications of tracheostomy were comparable between the groups, whereas the duration of ECMO and ICU length of stay increased significantly as the quartiles of tracheostomy timing increased. Patients in quartile 1 had the lowest hospital mortality rate (19.2%), whereas those in quartile 4 had the highest mortality rate (50.0%). Multivariate logistic regression analysis showed a significant association between the increment of the quartiles of tracheostomy timing and hospital mortality (adjusted odds ratio for quartile increment:1.55, 95% confidence interval 1.03–2.35, *p* for trend = 0.037).

**Conclusions:**

The timing of tracheostomy in patients requiring ECMO was significantly associated with patient outcomes in a time-dependent manner. Further investigation is warranted to determine the optimal timing of tracheostomy in terms of mortality.

**Supplementary Information:**

The online version contains supplementary material available at 10.1186/s40560-022-00649-w.

## Background

Tracheostomy is commonly performed in critically ill patients receiving mechanical ventilation. Early tracheostomy is being increasingly considered beneficial as it is well-established and provides various potential advantages in the management of mechanical ventilation. Tracheostomy for intubated patients decreases airflow resistance, breathing effort, and sedation requirement, while also improving patient comfort and mobility [1–3]. The effects of early tracheostomy in patients on mechanical ventilation have been studied comprehensively and have been shown to reduce the duration of treatment, mechanical ventilation, length of stay, and costs [4, 5]. Randomized controlled studies comparing early versus late tracheostomy have not established a causative effect on mortality [6–8]. However, several large observational studies have shown more favorable outcomes in patients undergoing early tracheostomies [9, 10]. A multicenter observational study of tracheostomized patients in the United States of America found that the mortality rate was 1.4 times higher in patients with late tracheostomy (≥ 14 days) versus early tracheostomy (< 14 days) [11]. Moreover, we have previously demonstrated a significant and incremental association between the timing of tracheostomy and mortality in a nationwide multicenter cohort study of critically ill patients on mechanical ventilation using the Japanese Intensive Care Patient Database [12, 13]. Thus, the benefits of early tracheostomy on patient outcomes, including mortality, have become more evident.

Extracorporeal membrane oxygenation (ECMO) is an important life-sustaining intervention for critically ill patients with cardiac or respiratory failure, which is refractory to conventional treatment. Patients receiving ECMO may require prolonged mechanical ventilation, and tracheostomy is often performed in these patients. The average or median duration of ECMO use ranges from 4 to 11 days, with patients reported to have been on ECMO for as long as 260 days [14, 15]. It is generally required that mechanical ventilation is continued during ECMO, and tracheostomy is performed in 18–51% of patients undergoing ECMO [16–18]. Recently, some studies have examined and reported on the procedures and complications associated with tracheostomy [19–22], however, there is a lack of evidence regarding the impact of tracheostomy timing on patient outcomes in patients receiving ECMO.

For ECMO recipients with high-severity and potentially life-threatening diseases, we recognized the need for a detailed study of tracheostomy timing and its association with improved patient outcomes. Accordingly, we aimed to investigate the association between tracheostomy timing and relevant clinical outcomes, including mortality, in critically ill patients receiving ECMO.

## Methods

### Conduct of the study and selection criteria

This retrospective cohort study was performed in the multidisciplinary intensive care unit (ICU) of a tertiary care hospital between April 2014 and December 2021. We included mechanically ventilated patients who underwent ECMO and tracheostomy during first ICU admission. Exclusion criteria included the following: (a) tracheostomy before ICU admission, and (b) subsequent ICU admission with ECMO.

This study was approved by the Ethics Review Board of Osaka University Hospital (Approval Number: 17471), and the need for consent for research participation was waived. The study was performed in accordance with the relevant guidelines and regulations.

### ICU setting and managements of patients

The 29-bed ICU admits to approximately 1000 critically ill patients annually and is fully capable of providing artificial life support, including ECMO. According to a closed model of care, intensivists direct aspects of patient care in collaboration with other primary team doctors. All patients were reviewed every morning by intensivists, primary team doctors, and bedside nurses to discuss patient-related issues and decisions for tracheostomy and management of mechanical ventilation, including weaning and extubation. Tracheotomy was performed in the operating room or ICU.

### Details of data collected

The characteristics collected for each patient included age, sex, body mass index (BMI), comorbidities (chronic heart failure, chronic respiratory failure, chronic liver disease, malignancy, immunodeficiency, and maintenance dialysis: [yes, no]), emergency admission (yes, no), Acute Physiology and Chronic Health Evaluation (APACHE) II scores as indicators of disease severity on ICU admission, coronavirus disease 2019 (COVID-19) infection (yes, no), diagnosis requiring ECMO intervention (heart failure [non-surgical], respiratory failure [non-surgical], cardiovascular surgery, thoracic surgery, or cardiac arrest), sternotomy (yes, no), and ECMO configuration (venovenous [VV] or venoarterial [VA]) performed. For patients on mixed modality ECMO, the main configuration was recorded. We also collected the following processes of care: renal replacement therapy (yes, no), use of steroids (yes, no), initiation of enteral nutrition, rehabilitation program (yes, no), initiation of rehabilitation program. Steroid use was defined as an equivalent of ≥ 20 mg methylprednisolone. The rehabilitation program included prescribed rehabilitation by occupational therapists. As for tracheostomy data, duration of mechanical ventilation before tracheostomy, type of tracheostomy (surgical or percutaneous), tracheostomy during ECMO (yes, no [indicating after ECMO removal]), and complications after tracheostomy (bleeding requiring blood transfusion or intervention, tracheal/esophageal injury, stoma infection, mediastinitis, pneumonia: [yes, no]) were collected. Patient outcomes, including duration of mechanical ventilation, occurrence of ventilator-associated pneumonia (VAP) (yes, no), liberation from mechanical ventilation during ICU stay (yes, no), duration of ECMO, length of ICU and hospital stay, ICU or hospital mortality (yes, no), patient status at hospital discharge (discharged home or to a nursing-care facility, transferred to other hospital, death), and mechanical ventilation on hospital discharge (yes, no) were recorded. Occurrence of VAP defined as clinical suspicion including ≥ two criteria (fever > 38.5 °C, leukocytosis > 10^9^/L or leukopenia < 4.10^8^/L, purulent secretions), a new or persistent infiltrate on chest radiography, and worsening oxygenation with confirmation by positive culture of a respiratory sample [23, 24]. Study follow-up began at the time of initiation of mechanical ventilation.

### Outcome measured

The primary outcome of this study was hospital mortality and the secondary outcome was ICU mortality.

### Statistical analyses

This study followed the Consolidated Standards of Reporting Trials (CONSORT) recommendations for reporting cohort studies (STROBE statement) [25]. To summarize the collected data, we calculated medians and interquartile ranges (IQRs) for continuous variables, and numbers and percentages for categorical variables. Patients were divided into four groups based quartiles of tracheostomy timing from the start of mechanical ventilation. Differences in proportions were evaluated using the Chi-square test or Fisher’s exact test, and differences in distributed data were evaluated using the Kruskal–Wallis test for the four groups. Univariable and multivariable logistic regression models were constructed to identify the relationships between the quartiles of tracheostomy timing and the primary and secondary outcomes. We described the crude and adjusted odds ratios (ORs) and their 95% confidence intervals (CIs) for each outcome per quartile. To adjust for confounding factors, age (1-year increment), sex (male, female), and the APACHE II score (1-point increment) were added to the model for hospital and ICU mortality. The nonlinear relationships between the timing of tracheostomy and the estimated hospital and ICU mortality were visually described using restricted cubic splines in the univariable logistic regression model. Moreover, we performed a subgroup analysis stratified by tracheostomy during ECMO. Multivariate logistic regression models were used to evaluate the interaction effect between the quartiles of tracheostomy timing and subgroups of hospital mortality. All statistical analyses were conducted using R version 4.0.4 (2021, R Foundation for Statistical Computing, Vienna, Austria) and Statistical significance was set at a two-sided *p*-value of 0.05.

## Results

### Patient characteristics

**Fig. 1:**
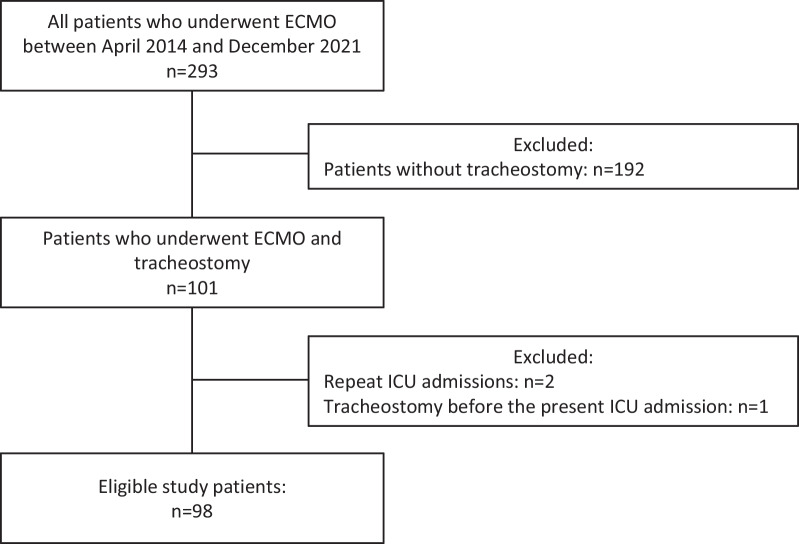
Patient inclusion flowchart. ECMO, extracorporeal membrane oxygenation; ICU, intensive care unit

A total of 7854 patients were admitted to the ICU during the study period, of which 293 required ECMO. Of these, 192 patients did not undergo tracheostomy and 101 patients who underwent ECMO and tracheostomy were assessed for eligibility. Additionally, three patients with repeat ICU admissions (*n* = 2) and tracheostomies on ICU admission (*n* = 1) were excluded. A total on 98 patients were thus eligible for analysis (Fig. 1). The study participants were divided into four groups according to the quartiles of tracheostomy timing: quartile 1 (≤ 15 days), quartile 2 (16–19 days), quartile 3 (20–26 days), and quartile 4 (> 26 days). Patient characteristics are shown in Table 1. Within the cohort, the median age was 58.5 [IQR, 45.5–74] years and 67 (68.4%) patients were male. With regard to ECMO configuration, 13 (13.3%) and 85 (86.7%) patients were on VV-ECMO and VA-ECMO, respectively. We found COVID-19 was more associated with patients in quartile 4 than in the other groups. The other patient characteristics were comparable between the groups.

**Table 1:** Baseline characteristics of the study cohort, stratified by quartile of tracheostomy timing

	Quartile 1 Tracheostomy ≤ 15 days (*n* = 26)	Quartile 2 Tracheostomy 16–19 days (*n* = 23)	Quartile 3 Tracheostomy 20–26 days (*n* = 25)	Quartile 4 Tracheostomy > 26 days (*n* = 24)	*p* value
Age, years	63.0 (49.0–75.8)	57.0 (49.0–76.5)	65.0 (52.0–75.0)	54.5 (15.0–64.5)	0.085
Sex, male	20 (76.9%)	19 (82.6%)	13 (52.0%)	15 (62.5%)	0.095
Body mass index, kg/m^2^	22.0 (20.5–24.6)	23.0 (18.7–26.3)	22.2 (20.5–24.7)	19.8 (18.1–22.9)	0.297
Comorbidity					
Chronic heart failure	6 (23.1%)	7 (30.4%)	8 (32.0%)	9 (37.5%)	0.759
Chronic respiratory failure	7 (26.9%)	5 (21.7%)	5 (20.0%)	3 (12.5%)	0.668
Chronic liver disease	0 (0%)	0 (0%)	1 (4.0%)	0 (0%)	0.735
Malignancy	0 (0%)	0 (0%)	0 (0%)	1 (4.2%)	0.480
Immunodeficiency	1 (3.8%)	4 (17.4%)	0 (0%)	2 (8.3%)	0.072
Maintenance dialysis	3 (11.5%)	1 (4.3%)	1 (4.0%)	2 (8.3%)	0.801
Emergency admission	23 (88.5%)	15 (65.2%)	21 (84.0%)	17 (70.8%)	0.176
APACHE II score	18.5 (17.0–22.8)	18.0 (14.5–21.0)	18.0 (14.0–21.0)	17.0 (12.5–20.0)	0.329
COVID-19 infection	0 (0%)	1 (4.3%)	0 (0%)	4 (16.7%)	0.016
Diagnosis for ECMO indication					
Heart failure (non-surgical)	4 (15.4%)	3 (13.0%)	7 (28.0%)	6 (25.0%)	0.459
Respiratory failure (non-surgical)	2 (7.7%)	2 (8.7%)	0 (0%)	4 (16.7%)	
Cardiovascular surgery	13 (50.0%)	14 (60.9%)	12 (48%)	12 (50.0%)	
Thoracic surgery	1 (3.8%)	2 (8.7%)	1 (4.0%)	1 (4.2%)	
Cardiac arrest	6 (23.1%)	2 (8.7%)	5 (20.0%)	1 (4.2%)	
Sternotomy	13 (50.0%)	15 (65.2%)	14 (56.0%)	14 (58.3%)	0.771
ECMO configuration					
VV	5 (19.2%)	3 (13.0%)	1 (4.0%)	4 (16.7%)	0.379
VA	21 (80.8%)	20 (87%)	24 (96%)	20 (83.3%)	

Data are presented as the median and interquartile range or as numbers (percentages)

*p* values are analyzed using the Chi-square (Fisher’s exact) test or the Kruskal–Wallis test

APACHE, Acute Physiology and Chronic Health Evaluation; COVID-19, coronavirus disease 2019; ECMO, extracorporeal membrane oxygenation; VV, venovenous; VA, venoarterial

### Details of tracheostomy and processes of care

All patients underwent surgical tracheostomy, and the proportion of patients who underwent tracheostomy during ECMO was similar in each group (Table 2). Although approximately 13% of all patients had bleeding complications, there were no significant differences in tracheostomy complications between the groups. The initiation of enteral nutrition, steroid use and prescriptions for rehabilitation were comparable among the four groups. However, in approximately 80% of patients who received rehabilitation, patients in quartile 4 had a significantly later initiation of rehabilitation, 41 (30–64) days after commencement of mechanical ventilation. The incidence of VAP was similar among the four groups. The patients in quartile 4 had a significantly longer duration of mechanical ventilation and ECMO compared to the other patient groups (70.6 [IQR, 46.9–103.4] and 31.5 [IQR, 13.0–59.3] days, respectively, *p* < 0.001 for both). Furthermore, the length of ICU stay of the patients was longest in quartile 4 at 79.6 (53.8–110.9) days (*p* < 0.001). In terms of patient status at hospital discharge, the patients in quartile 4 were least likely to be discharged home or nursing-care facility (12.5%) and had the highest mortality rate (50.0%). Across each group, 24.0–53.8% of patients were transferred to other hospitals. At hospital discharge, patients in quartile 4 were most frequently continued on mechanical ventilation (25.0%), with no statistically significant differences between the groups.

**Table 2:** Processes of care, tracheostomy data, and clinical outcomes

	Quartile 1 Tracheostomy ≤ 15 days (*n* = 26)	Quartile 2 Tracheostomy 16–19 days (*n* = 23)	Quartile 3 Tracheostomy 20–26 days (*n* = 25)	Quartile 4 Tracheostomy > 26 days (*n* = 24)	*p* value
Renal replacement therapy	17 (65.4%)	15 (65.2%)	19 (76.0%)	13 (54.2%)	0.471
Use of steroids	6 (23.1%)	4 (17.4%)	6 (24%)	9 (37.5%)	0.463
Initiation of enteral nutrition, day	3 (2–6)	4 (2–5)	3 (3–5)	5 (3–8)	0.472
Rehabilitation program	21 (80.8%)	18 (78.3%)	20 (80.0%)	17 (70.8%)	0.849
Initiation of rehabilitation program, day	23 (16–30)	25 (19–34)	32 (25–41)	41 (30–64)	0.002
Duration of mechanical ventilation before tracheostomy, days	9.0 (7.0–12.8)	17.0 (15.5–18.0)	22.0 (21.0–25.0)	39.5 (33.5–57.3)	< 0.001
Type of tracheostomy					
Surgical tracheostomy	26 (100%)	23 (100%)	25 (100%)	24 (100%)	0.977
Percutaneous tracheostomy	0 (0%)	0 (0%)	0 (0%)	0 (0%)	
Tracheostomy during ECMO	8 (30.8%)	10 (43.5%)	9 (36.0%)	8 (33.3%)	0.824
Complications after tracheostomy					
Bleeding requiring blood transfusion or intervention	4 (15.4%)	3 (13.0%)	2 (8.0%)	4 (16.7%)	0.845
Tracheal/esophageal injury	0 (0%)	0 (0%)	0 (0%)	0 (0%)	N/A
Stoma infection	0 (0%)	0 (0%)	0 (0%)	0 (0%)	N/A
Mediastinitis	1 (3.8%)	0 (0%)	0 (0%)	0 (0%)	1.000
Pneumonia	1 (3.8%)	3 (13.0%)	2 (8.0%)	1 (4.2%)	0.556
Duration of mechanical ventilation, days	27.6 (13.7–36.8)	33.0 (22.5–45.3)	38.1 (31.9–62.6)	70.6 (46.9–103.4)	< 0.001
Occurrence of VAP	9 (34.6%)	6 (26.1%)	9 (36.0%)	9 (37.5%)	0.849
Liberation from mechanical ventilation during ICU stay	16 (61.5%)	12 (52.2%)	11 (44.0%)	9 (37.5%)	0.365
Duration of ECMO, days	5.5 (3.0–17.5)	9.0 (7.0–20.5)	11.0 (10.0–39.0)	31.5 (13.0–59.3)	< 0.001
Length of ICU stay, days	30.8 (19.2–53.9)	35.7 (23.5–49.7)	43.5 (36.1–63.9)	79.6 (53.8–110.9)	< 0.001
Length of hospital stay, days	78.5 (52.5–100.8)	101.0 (59.5–239.5)	102.0 (59.0–206.0)	127.5 (88.8–214.3)	0.052
Patient status at hospital discharge					
Discharged home or to a nursing-care facility	7 (26.9%)	5 (21.7%)	11 (44.0%)	3 (12.5%)	0.086
Transferred to other hospital	14 (53.8%)	10 (43.5%)	6 (24.0%)	9 (37.5%)	
Death	5 (19.2%)	8 (34.8%)	8 (32.0%)	12 (50.0%)	
Mechanical ventilation on hospital discharge among survivor	2/21 (9.5%)	0/15 (0%)	1/17 (5.9%)	3/12 (25.0%)	0.181

Data are presented as the median and interquartile range or as numbers (percentages)

*p* values are analyzed using the Chi-square (Fisher’s exact) test or the Kruskal–Wallis test

ECMO, extracorporeal membrane oxygenation; ICU, intensive care unit; VAP, ventilator-associated pneumonia

### Relationship between the timing of tracheostomy and mortality

**Fig. 2:**
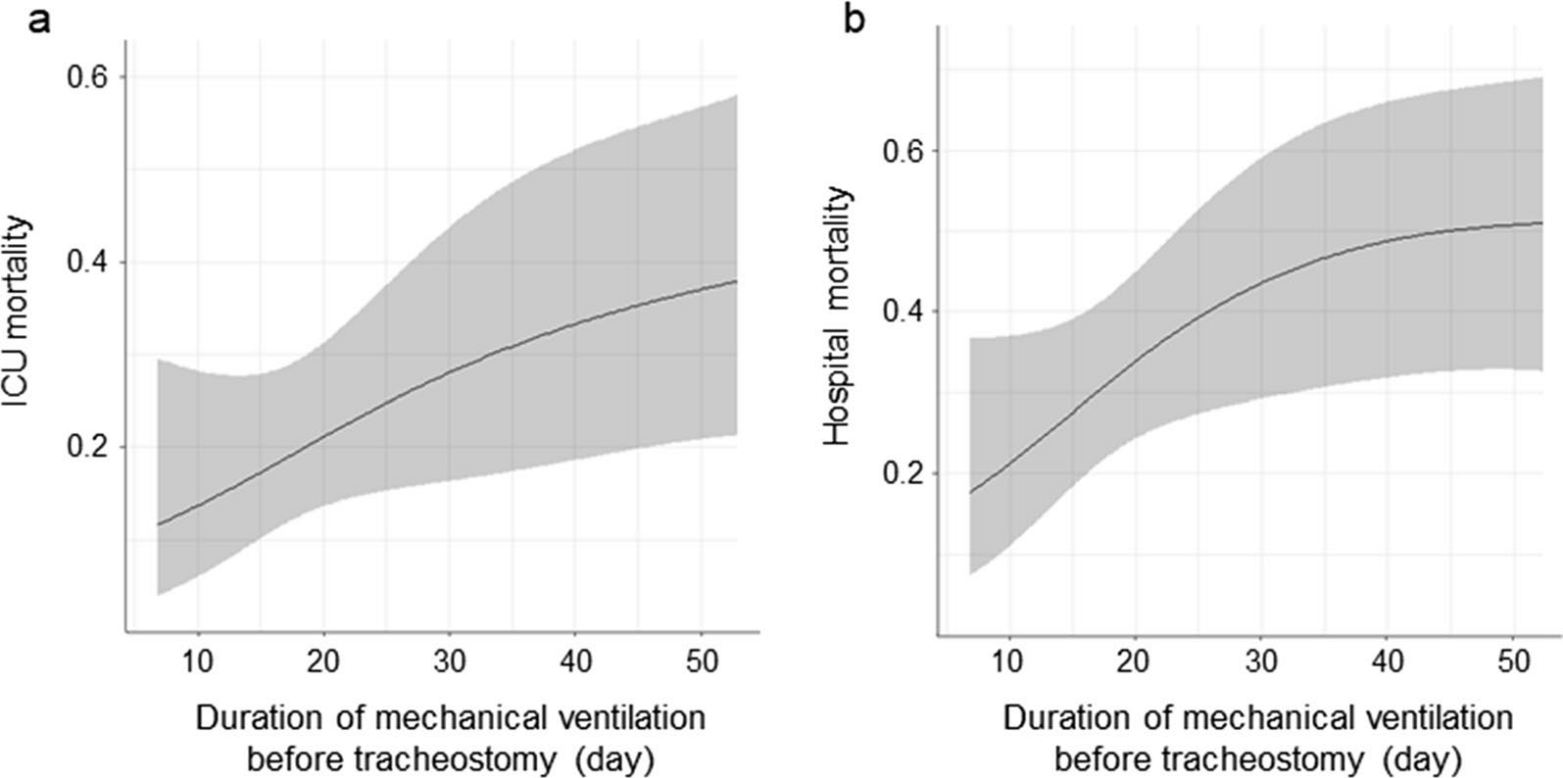
Mortality by the timing of tracheostomy. **a** ICU mortality; **b** hospital mortality. ICU, intensive care unit

The primary outcome, hospital mortality, increased as the quartiles of tracheostomy timing increased, with the highest rate in patients in quartile 4 (quartile 1, 19.2%; quartile 2, 34.8%; quartile 3, 32.0%; and quartile 4, 50.0%; *p* for trend = 0.037, Table 3). After adjusting for confounding factors, the adjusted proportion of hospital mortality also increased in a stepwise manner across increasing quartiles of tracheostomy timing (adjusted OR for quartile increment:1.55, 95% CI 1.03–2.35, *p* for trend = 0.037). The multivariable logistic regression analysis showed that the proportion of hospital mortality in patients in quartile 4 was significantly higher than that of patients in quartile 1 (adjusted odds ratio 4.39; 95% CI 1.16–16.60). In addition, subgroup analysis showed no significant interaction in terms of tracheostomy with or without ECMO (*p* = 0.359, Additional file 1). Among 35 patients who underwent tracheostomy during ECMO, hospital mortality was higher in patients in quartile 4 (crude OR: 3.00, 95% CI 0.36–24.90) than those in quartile 1 (OR for quartile increment: 1.34, 95% CI 0.70–2.54, *p* for trend = 0.374). In the 63 patients who underwent tracheostomy after ECMO removal, there was a significant trend toward a stepwise increase in hospital mortality as the quartiles of tracheostomy timing increased (OR for quartile increment: 2.04, 95% CI: 1.08–3.89, p for trend = 0.029). Furthermore, the association between mortality and timing of tracheostomy was consistently observed in the secondary outcome. Similarly, ICU mortality showed a progressive increase as the quartile of tracheostomy timing increased (adjusted OR for quartile increment,1.60; 95% CI 1.00–2.56, *p* for trend, 0.049). The ICU mortality rate was significantly higher in quartile 4 patients than in quartile 1 patients (adjusted OR: 3.41, 95% CI 0.82–14.20). Additionally, the restricted cubic spline showed that the unadjusted probabilities of hospital and ICU mortality significantly increased with the later timing of tracheostomy (Fig. 2).

**Table 3:** Association between quartile of tracheostomy timing and mortality

	Quartile 1 Tracheostomy ≤ 15 days (*n* = 26)	Quartile 2 Tracheostomy 16–19 days (*n* = 23)	Quartile 3 Tracheostomy 20–26 days (*n* = 25)	Quartile 4 Tracheostomy > 26 days (*n* = 24)	OR for quartile increment (95% CI)	*p* value for trend
ICU mortality						
* n* (%)	4 (15.4%)	3 (13.0%)	6 (24.0%)	9 (37.5%)		
Crude OR (95% CI)	1 (reference)	0.83 (0.16–4.15)	1.74 (0.43–7.09)	3.30 (0.86–12.70)	1.57 (1.01–2.46)	0.047
Adjusted OR (95% CI)^a^	1 (reference)	0.85 (0.16–4.39)	2.33 (0.53–10.20)	3.41 (0.82–14.20)	1.60 (1.00–2.56)	0.049
Hospital mortality						
* n* (%)	5 (19.2%)	8 (34.8%)	8 (32.0%)	12 (50.0%)		
Crude OR (95% CI)	1 (reference)	2.24 (0.61–8.21)	1.98 (0.55–7.16)	4.20 (1.19–14.80)	1.51 (1.03–2.23)	0.037
Adjusted OR (95% CI)^a^	1 (reference)	2.45 (0.65–9.22)	2.48 (0.65–9.53)	4.39 (1.16–16.60)	1.55 (1.03–2.35)	0.037

OR, odds ratio; CI, confidence interval; ICU, intensive care unit

^a^Adjusted OR for age, sex, APACHE II score in mortality

## Discussion

### Key findings

We conducted a retrospective observational study in patients who underwent ECMO and tracheostomy to assess how the timing of tracheostomy is related to clinical outcomes, including mortality. We found that approximately one-third of all ECMO patients required tracheostomy. In the comparison stratified by quartiles of the timing of tracheostomy, these differences did not translate into increased adverse events. Later timing of tracheostomy was associated with prolonged treatment duration, longer length of ICU stay, and increased mortality in a time-dependent manner.

### Relationship with prior studies

The use of ECMO has been intensifying for decades and has become available as a treatment option for critically ill patients. Indications, including pre-ECMO evaluations and management methods, have been well established; however, the mortality rate of patients requiring ECMO due to severe cardiac and respiratory failure were high and varied widely, ranging from 25 to 70% [26–31]. Tracheostomy has shown potential to improve patient outcomes by decreasing airway resistance; reducing the incidence of VAP through improved oral care and effective bronchopulmonary clearance; and promoting patient comfort, autonomy, and weaning of mechanical ventilation [1–3, 32, 33]. The benefits of early tracheostomy in patients requiring ECMO have been explored in several studies. A single-center, retrospective, observational study of 50 patients undergoing ECMO for acute respiratory distress syndrome was conducted in the United States of America [34]. DiChiacchio et al. compared early tracheostomy (≤ 7 days of ECMO commencement) and late tracheostomy (> 7 days), and found that hospital and 30-day mortality rates were similar between the two groups; however, the early tracheostomy group had a significantly shorter ECMO duration (median: 12 vs. 21 days) and reduced costs in relation to ECMO (median: $3624 vs. $5603). Tripathi et al. analyzed an international database of virtual pediatric systems (VPS, LLC). The authors evaluated 107 patients on postoperative cardiac ECMO who underwent tracheostomy and compared early tracheostomy (≤ 21 days from ICU admission) with late tracheostomy (> 21 days). Early tracheostomy was associated with more favorable patient outcomes, with significantly shorter ECMO duration (median: 8.0 vs. 12.9 days), ICU length of stay (median: 26.7 vs. 63.1 days), and duration of mechanical ventilation (median: 15.1 vs. 45.1 days). In our cohort, the incidence of VAP, which has been reported to have significant attribution to mortality in patients with ECMO [35], was comparable across the quartiles of tracheostomy timing. However, patients with early tracheostomy had started rehabilitation programs earlier, indicating earlier mobilization. The present study—with a detailed investigation wherein the timing of tracheostomy was divided by quartiles—revealed that early tracheostomy from the initiation of mechanical ventilation is significantly associated with favorable patient outcomes in terms of mortality, as well as reduced durations of mechanical ventilation, ECMO, and ICU length of stay.

Tracheostomy is one of the most widely used procedures in ICUs and may be performed at the bedside; however, complications such as bleeding and tracheal or bronchial injury can occur [36]. As ECMO circuits generally require systemic heparinization and may cause consumptive coagulopathy associated with blood–biomaterial interaction, bleeding complications are the predominant concern and may require temporary removal of the tracheostomy and oral reintubation for hemostasis [37, 38]. In the present study, we found that bleeding was the most common complication of tracheostomy, occurring in 13.3% of all patients. However, there was no difference in the incidence of complications according to the quartiles of tracheostomy timing. Additionally, 35 patients (35.7%) underwent tracheostomy during ECMO with no intergroup differences, and our subgroup analysis showed that early tracheostomy was associated with favorable patient outcomes similar to those in subgroups who received tracheostomy during or after ECMO. A recent single-center, retrospective, observational study of 54 patients receiving ECMO in the United States of America compared the complications of tracheostomy during ECMO with those after ECMO removal in detail [22]. Major complications, such as bleeding requiring intervention and aspiration, were equivalent; however, minor complications, including oozing and mucous plugging, were significantly more frequent in patients undergoing tracheostomy during ECMO (65.5 vs. 32.0%). In an international survey of 130 ECMO centers, 71.3% reported that they performed tracheostomy during ECMO, indicating that tracheostomy during ECMO was performed in practice [39]. However, with regard to tracheostomy during ECMO, the preponderance of the risks and benefits has not been identified, and careful individualized decisions should be made for each patient.

### Implications of study findings

Our findings imply that the timing of tracheostomy is significantly and gradually associated with patient outcomes, including mortality, in critically ill patients who require ECMO. Early tracheostomy from the commencement of mechanical ventilation was not accompanied by increased procedural complications and was independently associated with more favorable patient outcomes. The mortality rate of patients requiring tracheostomy and ECMO was as high as 33.7%. The findings of this study, which clarify the association between early tracheostomy (a simple and widely used procedure) and patient outcomes, are emerging insights that may provide a rationale for future investigations.

## Strengths and limitations

Our study has several strengths. Relatively few studies have been dedicated to the epidemiology of tracheostomy in critically ill patients requiring ECMO. The limited population of patients who underwent tracheostomy during their ICU admission was compensated with a detailed analysis by dividing the groups according to the quartiles of tracheostomy timing. Our study had the following limitations: first, this was a single-center, retrospective observational study with the inherent constraints including the challenge of generalizing across all geographic regions. However, it includes a wide variety of patients, including those with primary diseases and ECMO configurations. Moreover, tracheostomy implementation and mortality rates for patients requiring ECMO in this cohort were comparable to those in previous studies. Second, the tracheostomy procedure was not standardized, and potential confounding factors may not have been entirely evaluated. ECMO induction in this cohort was mostly performed by thoracic or cardiovascular surgeons, and surgical tracheostomy was performed in all tracheostomy procedures. Furthermore, the decision for late tracheostomy implementation was multifactorial. Of the patients in quartile 4, 16 patients performed tracheostomy a median of 17.5 days after ECMO removal, aside from the 8 patients who had tracheostomy during ECMO (Table 2). The main reasons for the delay were: coagulopathy in 4 patients, infection in 4 patients, extubation failure in 3 patients, concern for mediastinitis after sternotomy in 2 patients, awaiting negative polymerase chain reaction for COVID-19 in 2 patients, and airway obstruction in 1 patient. Third, the associations described in the observational studies might be overestimated due to immortal time bias [40]. Immortal time is the period of time during which the patients in a cohort study cannot experience the study outcome (death) between the beginning of the study observation and the end of follow-up. Immortal time can arise when the study comparison is based on the groups divided by the exposure that is observed after the patient enters the study. In the present study based on the tracheostomy timing, patients who underwent tracheostomy later may have experienced longer duration of mechanical ventilation and hospitalization, which are relevant to patient outcomes. There is a possibility of biased results towards worse outcomes for late tracheostomy.

## Conclusion

In our study of critically ill patients requiring ECMO, the timing of tracheostomy was significantly and time-dependently related to patient outcome. Early tracheostomy is potentially advantageous in terms of mortality, and further studies are warranted to identify the optimal timing of tracheostomy in patients requiring ECMO.

## Supplementary Information

**Additional file 1. **Subgroup analysis for hospital mortality for each quartile of tracheostomy timing.

## Data Availability

The datasets generated and/or analyzed during the current study are available from the corresponding author upon reasonable request.

## Abbreviations

APACHE: Acute Physiology and Chronic Health Evaluation
BMI: Body mass index
CI: Confidence interval
COVID-19: Coronavirus disease 2019
ECMO: Extracorporeal membrane oxygenation
ICU: Intensive care unit
IQR: Interquartile ranges
OR: Odds ratio
VA: Venoarterial
VAP: Ventilator-associated pneumonia
VV: Venovenous
