# Supplementary material for: Timing of tracheostomy and patient outcomes in critically ill patients requiring extracorporeal membrane oxygenation: a single-center retrospective observational study
Source: J Intensive Care. 2022 Dec 30;10:56. doi: 10.1186/s40560-022-00649-w (PMC9802016; doi:10.1186/s40560-022-00649-w)
Supplement: Supplementary file 1 — Additional file 1. Subgroup analysis for hospital mortality for each quartile of tracheostomy timing. [file 40560_2022_649_MOESM1_ESM.docx]

**Additional file 1.** Subgroup analysis for hospital mortality for each quartile of tracheostomy timing

|  | Quartile 1  Tracheostomy  ≤15 days  (n=26) | Quartile 2  Tracheostomy  16–19 days  (n=23) | Quartile 3  Tracheostomy  20–26 days  (n=25) | Quartile 4  Tracheostomy >26 days  (n=24) | OR for quartile  increment  (95% CI) | P value for trend | P value for interaction* |
| --- | --- | --- | --- | --- | --- | --- | --- |
| Tracheostomy during  ECMO (n=35) |  |  |  |  |  |  | 0.359 |
| n (%) | 4 (50.0%) | 6 (60.0%) | 5 (55.6%) | 6 (75.0%) |  |  |  |
| Crude OR (95% CI) | 1 (reference) | 1.50  (0.23–9.80) | 1.25  (0.19–8.44) | 3.00  (0.36–24.90) | 1.34  (0.70–2.54) | 0.374 |  |
| Tracheostomy after  ECMO removal (n=63) |  |  |  |  |  |  |  |
| n (%) | 1 (5.6%) | 2 (15.4%) | 3 (18.8%) | 6 (37.5%) |  |  |  |
| Crude OR (95% CI) | 1 (reference) | 3.09  (0.25–38.30) | 3.92  (0.37–42.20) | 10.20  (1.07–97.40) | 2.04  (1.08–3.89) | 0.029 |  |
| * P value for interaction is calculated in terms of tracheostomy with or without ECMO.  ECMO, extracorporeal membrane oxygenation; OR, odds ratio; CI, confidence interval | | | | | | | |
